# Ultrafast synthesis of L-His-Fe_3_O_4_ nanozymes with enhanced peroxidase-like activity for effective antibacterial applications

**DOI:** 10.3389/fbioe.2025.1548025

**Published:** 2025-03-28

**Authors:** Ye Yuan, Yuan Liu, Zhipeng Shen, Huidan Wu, Lantian Meng, Xiaoxiao Guo, Bing Jiang, Ling Fang

**Affiliations:** ^1^ Nanozyme Laboratory in Zhongyuan, School of Basic Medical Sciences, Zhengzhou University, Zhengzhou, China; ^2^ Nanozyme Laboratory in Zhongyuan, Henan Academy of Innovations in Medical Science, Zhengzhou, Henan, China; ^3^ Department of Dermatology, Xishan People’s Hospital of Wuxi City, Wuxi Branch of Zhongda Hospital Southeast University, Wuxi, Jiangsu, China; ^4^ Jiangsu Institute of Parasitic Diseases, Wuxi, Jiangsu, China

**Keywords:** nanozyme, antibacterial, l-histidine, Fe_3_O_4_, biofilm

## Abstract

**Background:** Bacterial resistance remains a significant challenge, necessitating the development of new antibacterial strategies. This study introduces a rapidly synthesized L‐histidine‐ Fe_3_O_4_ (L‐His‐Fe_3_O_4_) nanozyme with enhanced peroxidase (POD)‐like activity, designed to improve antibacterial efficacy and accelerate the healing of bacteria-infected wounds.

**Methods:** We successfully synthesized L‐His‐Fe_3_O_4_ using an ultrafast, room-temperature synthesis method, and observed its anti-infection effect and explored its anti-infection mechanism through *in vivo* and *in vitro* antibacterial experiments.

**Results:** We produced L‐His‐Fe_3_O_4_ cost-effectively while preserving L‐His, which was essential for its catalytic and antibacterial functions. The resulting nanozyme demonstrated exceptional antibacterial activity against both Gram-positive (*S. aureus*) and Gram-negative (*E. coli*) bacteria. *In vivo* experiments revealed that L‐His‐Fe_3_O_4_ outperformed vancomycin in reducing bacterial viability and effectively promoting wound healing, all while maintaining excellent biosafety with no adverse effects on blood or liver functions.

**Discussion:** These findings highlight the potential of L‐His‐Fe_3_O_4_ for large-scale production and practical use in treating bacterial infections, offering a promising approach to combating antibiotic-resistant pathogens.

## 1 Introduction

The misuse and overuse of antibiotics have led to an alarming rise in multidrug-resistant bacteria, posing a severe threat to global public health (Brown and Wright, 2016; Pogue et al., 2015). Infections such as tuberculosis and gonorrhea are increasingly challenging to treat due to this resistance (Alim-Marvasti et al., 2016; Lee et al., 2020). Unfortunately, the development of new antibiotics has lagged behind the rapid emergence of bacterial resistance (Theuretzbacher et al., 2020; Brussow, 2024; Burki, 2018). The World Health Organization (WHO) predicts that by 2050, antibiotic resistance could result in up to 10 million deaths annually, identifying it as one of the top ten global threats to humanity (He et al., 2014). To address bacterial resistance, researchers have explored various novel antibacterial strategies (Panda et al., 2018; Gupta et al., 2019; Li et al., 2019; Makabenta et al., 2021), including antimicrobial peptides, bacteriophages, probiotics, and plant extracts; however, much of this research remains in its early stages.

In recent years, advancements in nanotechnology have led to the application of nanomaterials in medicine (Fan et al., 2021; Wang et al., 2018; Seaberg et al., 2023; Gao et al., 2007). In 2007, the Yan group demonstrated that Fe_3_O_4_ possesses peroxidase-like activity (Gao et al., 2024; Xie et al., 2024), paving the way for the development of nanozymes-nanomaterials with enzyme-like catalytic properties (Jiang et al., 2019; Sheng et al., 2024). Due to their adjustable physicochemical properties and unique catalytic activities, nanozymes hold significant promise for antibacterial applications (Liu et al., 2024). They can effectively kill bacteria by generating reactive oxygen species (ROS), such as hydroxyl radicals (·OH) and superoxide anions (·O_2_^−^) (Song et al., 2024; Trujillo-Alonso et al., 2019). Numerous studies have reported various nanozymes for antibacterial purposes, with Fe_3_O_4_ receiving FDA approval for biomedical applications, highlighting its high biosafety profile (Wei et al., 2021). Consequently, Fe_3_O_4_ emerges as a particularly promising candidate in combating bacterial infections. However, the primary synthesis methods for Fe_3_O_4_ like hydrothermal and solvothermal approaches are often tedious, time-consuming, expensive, and high temperature, which hinders large-scale production (Dong et al., 2022; Liu et al., 2021). Thus, there is an urgent need to improve Fe_3_O_4_ synthesis methods to accelerate the development of new antibacterial agents.

In this work, we present a low-cost and ultrafast method for synthesizing Fe_3_O_4_ to enhance its antibacterial properties. The synthesis of Fe_3_O_4_ at room temperature can preserve the structure and biological function of the modified histidine (L-His), thereby enhancing its antibacterial properties. In detail, by mixing ferrous chloride and L-His in an ammonium hydroxide solution for only 1 minute, we successfully synthesized L-His-Fe_3_O_4_. This method leverages histidine’s ability to enhance peroxidase (POD)-like activity through its activation of hydrogen peroxide reduction reactions (Yang et al., 2019; Du et al., 2022). Notably, this reaction occurs at room temperature, preserving the functional integrity of L-His. The improved POD-like activity of L-His-Fe_3_O_4_ catalyzed the production of hydroxyl radicals (·OH), which disrupt bacterial cell membranes and degrade biofilms. Both *in vitro* and *in vivo* antibacterial tests demonstrated the strong antimicrobial efficacy of L-His-Fe_3_O_4_. In wound healing studies, L-His-Fe_3_O_4_ exhibited a superior capacity to promote healing compared with vancomycin. Additionally, L-His-Fe_3_O_4_ showed excellent biosafety, reinforcing its potential as a novel antibacterial agent. Moreover, the synthesis strategy of L-His-Fe_3_O_4_ at room temperature provide a new pathway for complete modification of different functional groups of Fe_3_O_4_.

## 2 Materials and methods

### 2.1 Chemical reagents, bacteria, and animals

Ferrous chloride (FeCl_2_) and L-His were purchased from Sigma and Beyotime, respectively. Ammonium hydroxide was obtained from J&K Scientific. MRSA (ATCC 43300) and *S. aureus* (ATCC 29213) were sourced from the American Type Culture Collection (ATCC), and *E. coli* (CCUG58541) was obtained from the BeNa Culture Collection (BNCC). Balb/c mice were purchased from Qinglongshan Laboratory Animal Center. Fresh O-type red blood cells from Wuxi Central blood bank. Yeast extract (LP0021), glucose, and starch were obtained from Oxoid (UK). Dimethyl sulfoxide (DMSO) and agar were purchased from Sangon Biotech (China). Cell Counting Kit-8, crystal violet, and hydrogen peroxide (H_2_O_2_) were acquired from Aladdin Chemistry (Shanghai, China). Propidium iodide (PI) and Reactive Oxygen Species Assay Kit were sourced from Beyotime Biotechnology (Shanghai, China), while SYTO (Panda et al., 2018) green fluorescent nucleic acid stain was obtained from Thermo Fisher Scientific (Waltham, MA).

### 2.2 Theoretical calculation

Calculations were performed using the CP2K 2024.1 program. In the geometry optimization, Basis Set Superposition Error (BSSE) correction, and single-point calculations, the PBE-D3(BJ) functional was employed, incorporating Grimme’s dispersion correction (D3) and the Becke-Johnson (BJ) damping factor. The DZVP-MOLOPT-SR-GTH basis set and the TZVP-MOLOPT-SR-GTH basis set were used for optimization and single-point calculations, respectively. Optimization continued until atomic forces were less than 0.02 eV/Å, with the self-consistent electronic energy convergence criterion set at 10^−6^ eV. Multiwfn 3.8 Dev was used for computational analysis, and visualization was performed with VESTA and Visual Molecular Dynamics (VMD) 1.9.3.

### 2.3 Bacterial strains and growth conditions

*S*. *aureus* and *E. coli* were cultured in LB medium, composed of 10 g of tryptone, 5 g of yeast extract, 10 g of sodium chloride, and 15–20 g of agar powder for solid medium. Agar plates and liquid cultures were incubated at 37°C, and frozen stocks of strains were stored at −80°C in LB containing 30% (v/v) glycerol.

### 2.4 *In Vitro* antibacterial assays

A colony of *S*. *aureus* or *E. coli* was randomly selected from LB agar plates, inoculated into 5 mL of LB culture, and incubated at 37°C for 12–18 h. After 24 h, the required amount of *S. aureus* or *E. coli* bacterial solution was transferred into fresh medium at a 1:100 ratio and cultured at 37°C for 4–6 h. When the OD_600_ reached 0.5, 100 μL of bacterial inoculum was mixed with H_2_O (900 μL) as the control, while the experimental groups included L-His-Fe_3_O_4_, L-His-Fe_3_O_4_ + H_2_O_2_, and H_2_O_2_. After incubation at 37°C for a specified time, bacterial viability was assessed by plating bacteria with appropriate dilution and calculating the colony-forming units per mL (CFU/mL). The fluorescent probe DCFH-DA was used to assess lipid peroxidation levels in the bacteria.

### 2.5 Observation by confocal laser microscopy (CLSM)

To facilitate observation of *S*. *aureus* biofilm changes, biofilms were cultured on a specialized cell climbing plate in a 24-well format, following previous *S. aureus* biofilm culture methods. Tryptone soy broth (TSB) was used as the medium. The procedure is as follows.

(1) A sterile cell pad was placed at the bottom of a well in a 24-well plate, and 2.8 mL TSB culture solution was added and incubated for 30 min before discarding the TSB.
(2) *S. aureus* solution was diluted with TSB to an OD_600_ of 0.5 at a 1:100 ratio, with 2.5 mL added per well, and incubated at 37°C for 24 h. To maintain a humid environment conducive to biofilm growth, saline was added to the remaining empty wells.
(3) Once the biofilm matured, the specified treatment substance was added. The culture solution and non-adherent bacteria were gently removed, and each well was washed twice with 2.5 mL PBS. The treatment was then applied for 3 h.
(4) For laser confocal observation, bacterial cells within the biofilm were stained with SYTO9/PI using a double-labeled fluorescence method and observed under a confocal laser microscope (SYTO9 = 0.5 µM; PI = 1 µM).

### 2.6 Inner and outer membrane permeability test

8-aniline-1-naphthalenesulfonic acid (ANS) has a non-polar benzene ring, which binds to the membrane lipid at the lipid-water junction. The fluorescence intensity of ANS is related to the fluidity of the polar region of the membrane lipid. Membrane permeability was assessed using the ANS uptake method. A solution of 20 µM ANS and *S. aureus* (10^7^ CFU/mL) was prepared in 0.9% NaCl and incubated in darkness for 30 min. After centrifugation at 5000 g for 5 min, samples were washed and re-suspended in 0.9% NaCl. Fluorescence emission was measured at 450–600 nm with excitation at 380 nm. 3, 3′- Dipropylthiadicarbocyanine Iodide [diSC3(5)], a (C3) short alkyl tail carbonyl cyanine dyes, this kind of cationic dye can be used to detect and measure caused by membrane modification reagent across membrane potential or structural changes. Depolarization measurements were performed using diSC3(5) uptake with similar processing steps [diSC3(5), 4 μM; 622 nm excitation, 670 nm emission].

### 2.7 TEM (transmission electron microscope) and SEM (scanning electron microscope)

For each group, 0.5 mL of bacterial suspension (1.0 × 10^9^ CFU/mL) was added to a 1.5 mL aseptic centrifuge tube along with 0.5 mL of ultra-pure water, H_2_O_2_, or L-His-Fe_3_O_4_ + H_2_O_2_. Each group’s bacterial solution was incubated at 37°C for 3 h. After incubation, samples were centrifuged and washed twice with ultra-pure water. The bacterial precipitate was then mixed with 2.5% glutaraldehyde solution and fixed at 4°C for 24 h. Bacterial morphology was observed and photographed using TEM and SEM.

### 2.8 Crystal violet staining

#### 2.8.1 Cell culture

*S. aureus* solution with OD_600_ = 0.5 was diluted with biofilm culture medium (TSB) at a ratio of 1:100, with 100 µL added per well. Cultures were incubated at 37°C with 5% CO_2_ for 24 h.

#### 2.8.2 Add test compound

A medium containing the appropriate concentration of the test compound was added, and the cell culture plate was incubated for the specified time. Rinsing to remove floating bacteria: Methanol was used to rinse off floating bacteria, followed by a 15-min fixation with methanol, samples were then air-dried before staining.

#### 2.8.3 Crystal violet

1 mL of 0.1% crystal violet solution was added to each well and stained for 15 min. Excess stain was rinsed off with distilled water. Dissolution of Crystal Violet: 33% glacial acetic acid was added to dissolve the crystal violet.

### 2.9 *In vitro* safety evaluation

HaCaT cells were inoculated into 96-well plates at a density of 5 × 10^3^ cells per well and cultured in a serum-containing medium for 24 h (37°C, 5% CO_2_). Culture medium containing varying concentrations of L-His-Fe_3_O_4_ (0, 100, 200, 400, 500 μg/mL) was then applied for 24 and 48 h. The medium was discarded, cells were washed once with PBS, and then cultured with CCK-8 for 3–4 h. After incubation, the 96-well plates were centrifuged (1,000 rpm for 5 min), 100 μL of supernatant was pipetted, and absorbance was measured at 490 nm.

### 2.10 Red blood cell hemolysis test

Fresh O-type red blood cells from Wuxi Central blood bank (Ethics Committee of Jiangsu Institute of Parasitic Diseases: JIPD-2022-005). An appropriate amount of human red blood cells was obtained and diluted fivefold with PBS buffer to prepare a red blood cell suspension. A 20 μL aliquot of the diluted suspension was mixed with different concentrations of L-His-Fe_3_O_4_. Triton X-100 and PBS buffer were used as positive and negative controls, respectively. All samples were incubated at 37°C for 2 h, then centrifuged at 1,000 rpm for 5 min. Photos were taken, and the supernatant was transferred to a 96-well plate for absorbance measurement at 540 nm.

### 2.11 *In vivo* safety assessment

Mouse wounds were treated externally with L-His-Fe_3_O_4_ once daily for seven consecutive days. After this period, histopathological sections of major organs (heart, liver, spleen, lung and kidney) were prepared for HE staining and photographed under the bright-field mode of a fluorescence microscope.

### 2.12 Animal experiment

Male Balb/c mice (20–25 g, 6–7 weeks of age) were randomly divided into four groups (n = 8), consisting of (1) control, (2) vancomycin (2 mg/mL, 20 μL), (3) hydrogen peroxide, and (4) L-His-Fe_3_O_4_ + H_2_O_2_ (2 mg/mL, 20 μL). After shaving, mice were anesthetized, and a circular wound approximately 0.8 cm in diameter was created on the back using surgical scissors. MRSA (1.0 × 10^8^ CFU/mL) was then applied. After 24 h, an animal model of wound infection was established. Treatments were administered daily for the first 7 days following infection. On the 7th day, three mice from each group were randomly selected, wound tissue was collected, ground, diluted, and plated to observe *S. aureus* counts by plate counting. Photos of wounds were taken on days 1, 3, 5, 7, 9 and 11 to document changes and measure wound size. Mice were euthanized, and organs and wound tissues were collected for hematoxylin-eosin (H&E) staining and Masson analysis.

## 3 Results

### 3.1 The synthesis and characterization of L-His-Fe_3_O_4_

**FIGURE 1:**
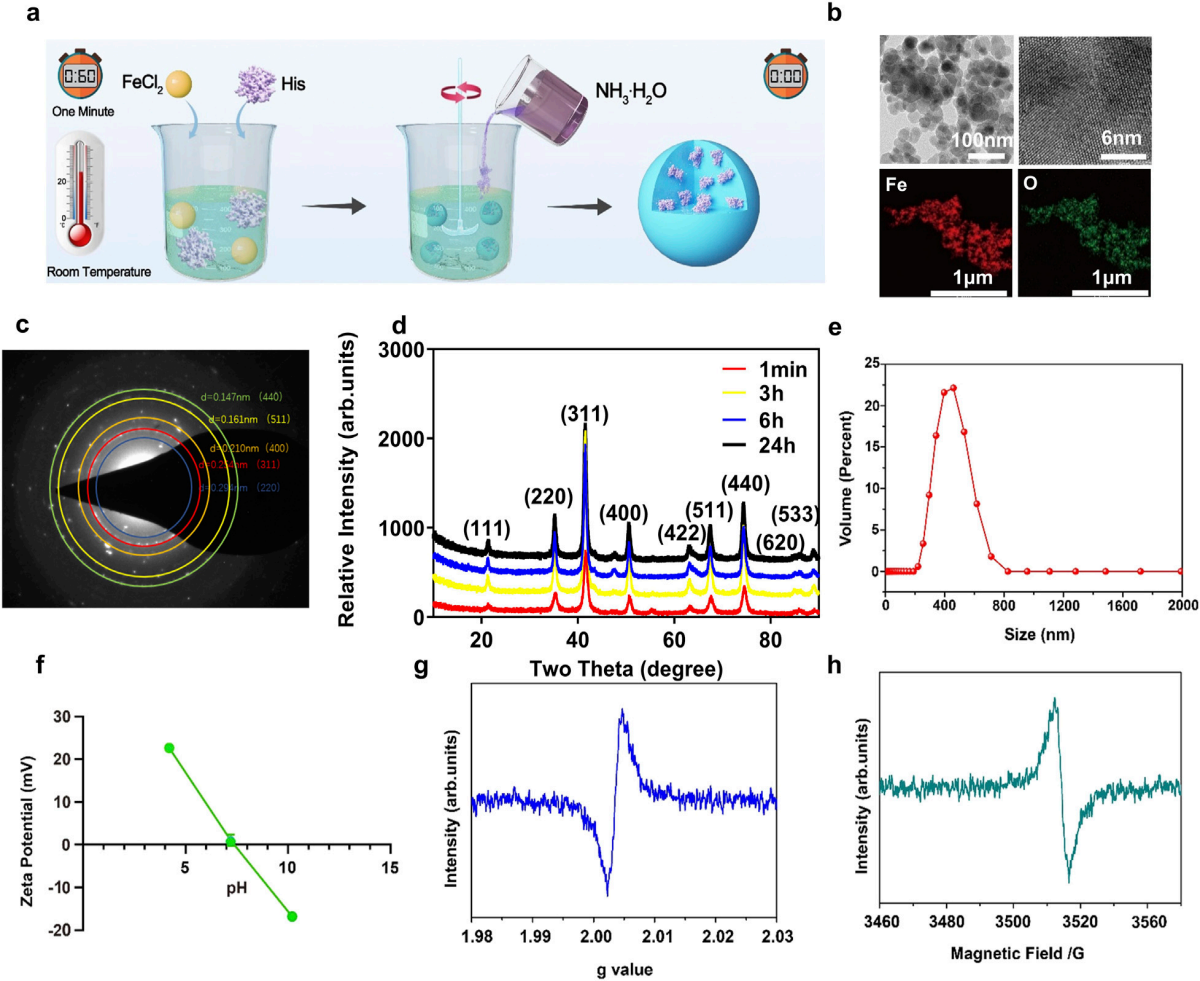
Synthesis and characterization of L-His-Fe_3_O_4_. **(a)** The preparation process of L-His-Fe_3_O_4_. **(b)** TEM image and the corresponding element mapping image. **(c)** HAADF-STEM image. **(d)** XRD spectrums. **(e)** Size distribution. **(f)** Zeta potentials at different pH conditions. **(g, h)**. The EPR spectra of L-His-Fe_3_O_4_.

L-His-Fe_3_O_4_ was synthesized by stirring at room temperature for 1 min. Briefly, ferrous chloride and L-His solutions were mixed, followed by the addition of 28% ammonium hydroxide. After stirring at room temperature for 1 min and drying at 60°C, L-His-Fe_3_O_4_ was obtained (Figure 1a). The yield of L-His-Fe_3_O_4_ is 36.48%, the morphology of L-His-Fe_3_O_4_ was observed by transmission electron microscopy (TEM). As shown in Figure 1b, TEM images demonstrated spherical particles of L-His-Fe_3_O_4_ with a diameter of approximately 20 nm, primarily composed of Fe and O elements. The selected area electron diffraction (SAED) pattern (Figure 1c) of L-His-Fe_3_O_4_ confirms the apparent diffraction rings, which correspond to the (440) (511) (400), and (311) crystal planes. To confirm the efficiency of the synthesis method, X-ray diffraction (XRD) analysis was used to monitor the crystal phase at different reaction times. As shown in Figure 1d, the diffraction peaks of the product at 1 min aligned well with those of Fe_3_O_4_ (PDF#19–0,629). After 3, 6, and 12 h of reaction, the crystal phase remained unchanged, indicating the high efficiency of the synthesis method. The particle size distribution of L-His-Fe_3_O_4_ ranged from 200 to 800 nm (Figure 1e), and the zeta potential varied with changes in pH (Figure 1f). The generation of oxygen vacancies (OVs) in L-His-Fe_3_O_4_ was measured by electron paramagnetic resonance (EPR). The g = 2.003 and the peak with magnetic field at around 3,500 revealed that L-His-Fe_3_O_4_ possessed at ypical signal of Ovs (Figures 1g, h).

### 3.2 Enzyme activity characterization and theoretical calculation

**FIGURE 2:**
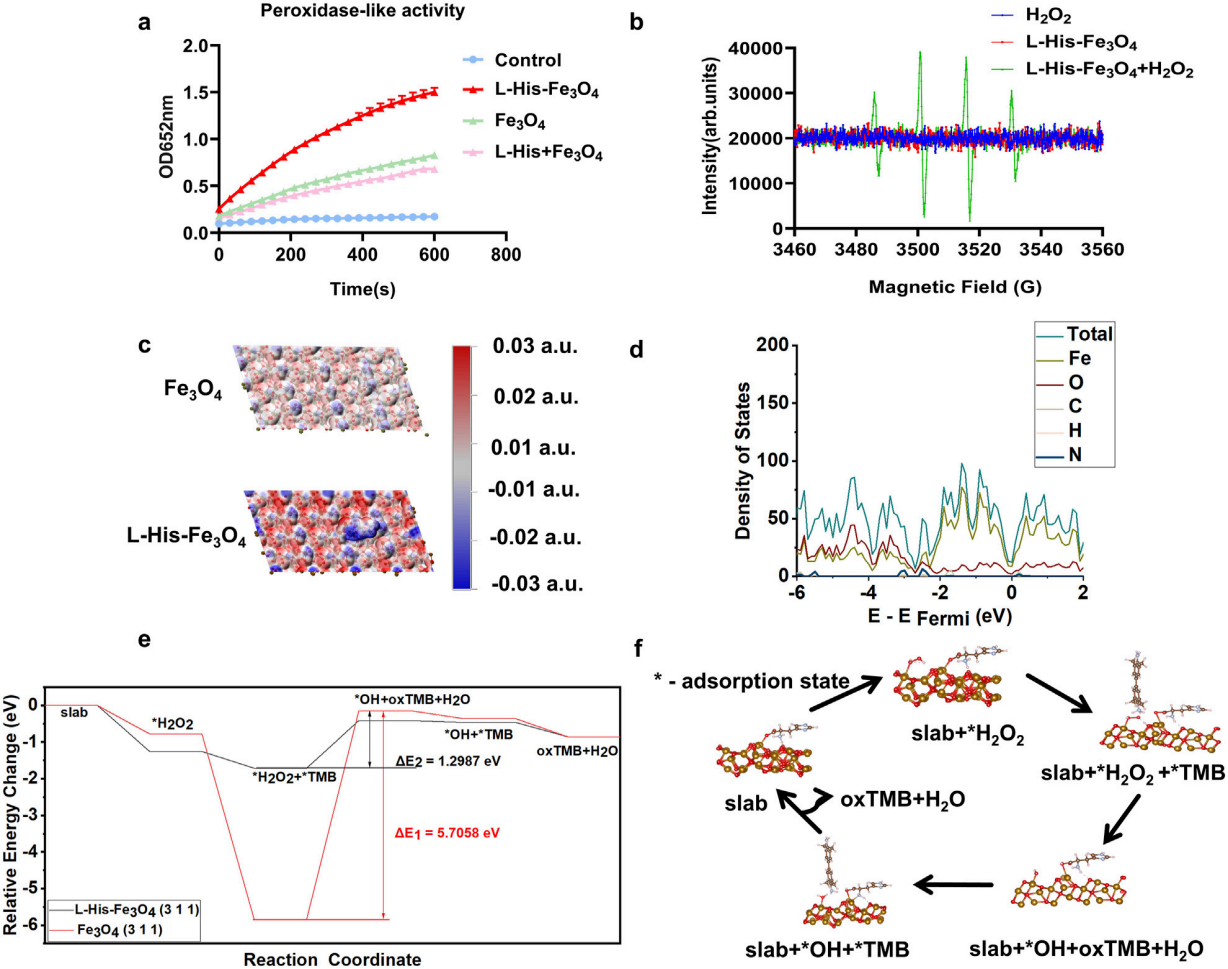
Enzyme activity characterization and theoretical calculation of L-His-Fe_3_O_4_. **(a)** Reaction-time curves for the TMB colorimetric reaction catalyzed by Fe_3_O_4_ and L-His-Fe_3_O_4_. **(b)** ESR spectra of hydroxyl radicals generated by H_2_O_2_, Fe_3_O_4_, and L-His-Fe_3_O_4_ + H_2_O_2_. **(c)** Electrostatic potential analysis of Fe_3_O_4_ and L-His-Fe_3_O_4_. **(d)** Density of states diagram of L-His-Fe_3_O_4_. **(e)** Energy changes during the reaction process of L-His-Fe_3_O_4_. **(f)** Reaction pathway diagram of L-His-Fe_3_O_4_.

To assess the peroxidase -like activity of L-His-Fe_3_O_4_, the absorption intensity of the L-His-Fe_3_O_4_ nanozyme-catalyzed TMB colorimetric reaction at 652 nm was measured (Figure 2a). The absorbance intensity of oxidized TMB (ox-TMB) increased over time. Furthermore, compared with Fe_3_O_4_, L-His-Fe_3_O_4_ exhibited higher POD-like activity. The generation of ∙OH radicals was monitored using electron spin resonance (ESR) spectroscopy, and the results (Figure 2b) showed a significant increase in ∙OH signal intensity, indicating that L-His-Fe_3_O_4_ could effectively catalyze the formation of ∙OH from H_2_O_2_.

To investigate L-His’s role in the catalytic reduction of H_2_O_2_ from an electronic structure perspective, we conducted electrostatic potential analysis, density of states analysis, and differential charge density mapping before and after L-His modification of the surface. Electrostatic potential analysis on the Fe_3_O_4_ (311) surface was performed before and after L-His adsorption (Figure 2c). Red regions indicate positive electrostatic potential, while blue regions indicate negative electrostatic potential. These results demonstrate that the modification increased the polarity of the Fe_3_O_4_ surface, facilitating the reaction between Fe_3_O_4_ and H_2_O_2_ during TMB oxidation.

Density of states plots for the slab model of the Fe_3_O_4_ (311) surface before and after L-His adsorption is shown in Figures 2d, e. The results indicate that L-His modification led to a more continuous distribution of states near the Fermi level in Fe_3_O_4_, enhancing electron transfer during the catalytic process, reducing the energy barrier, and promoting the reaction (Supplementary Figure S1). Differential charge density maps for H_2_O_2_ adsorption on the Fe_3_O_4_ (311) surface before and after L-His modification is presented in Supplementary Figure S2. The modification increased the interaction between the Fe_3_O_4_ surface and H_2_O_2_, with differential charge density results also indicating enhanced charge exchange between the modified Fe_3_O_4_ (311) surface and H_2_O_2_. According to Coulomb’s law, this intensifies the interaction between the two.

To evaluate the impact of modification on Fe_3_O_4_’s catalytic process in the oxidation of TMB by H_2_O_2_, we calculated the energy changes throughout the reaction, as shown in Figures 2e, f. Steps one to six correspond to the stages in the reaction pathway diagram, with “*” denoting the adsorption state on the catalyst surface. Oxidized TMB (oxTMB) refers to TMB in its oxidized form after hydrogen loss (Figure 2f) (Equations 1-6).

E1=Eslab=0 (1)

$$E2=E*H_{2}O_{2}$$ (2)

$$E3=E*H_{2}O_{2}+*\text{TMB}$$ (3)

$$E4=E*\text{OH}+*\text{oxTMB}+H_{2}O$$ (4)

E5=E*OH+* oxTMB (5)

$$E6={\text{EH}}_{2}O+*\text{oxTMB}$$ (6)

The calculations revealed that: (1) Compared to the unmodified Fe_3_O_4_ surface, the binding energy of L-His-modified Fe_3_O_4_ with H_2_O_2_ is more negative, indicating a stronger interaction between the modified Fe_3_O_4_ surface and H_2_O_2_, which facilitates H_2_O_2_ activation during catalysis; (2) The energy barrier during the reaction process, occurring in the reduction of H_2_O_2_ to ?OH by TMB, was significantly lowered from 5.7058 eV to 1.2987 eV with L-His modification, greatly enhancing the reaction progress.

### 3.3 The anti-bacterial activity of L-His-Fe_3_O_4_ against *S. aureus* and *E. coli*

**FIGURE 3:**
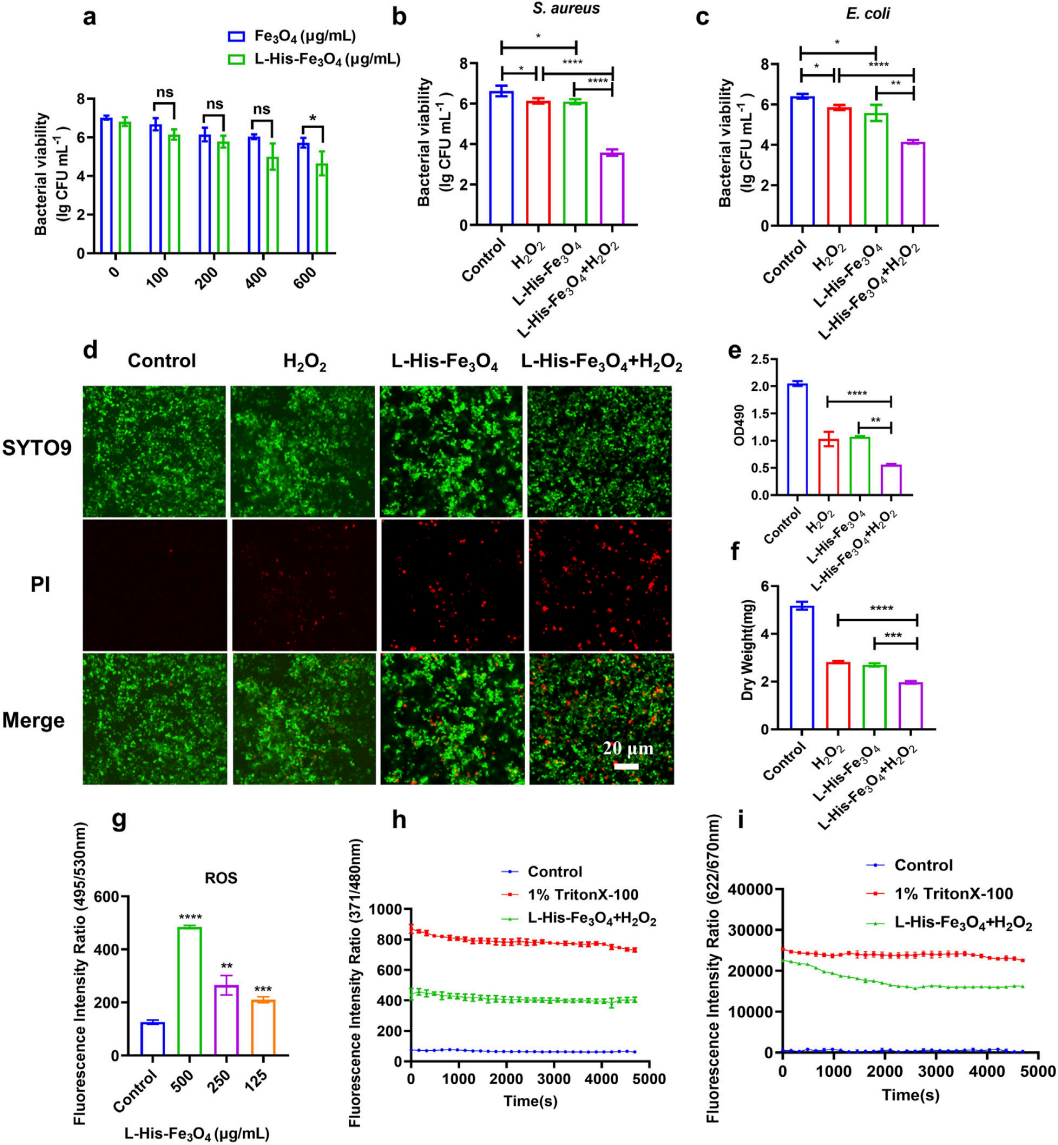
Test of antibacterial property and bactericidal mechanism of L-His-Fe_3_O_4_. **(a)** Measurement of *S. aureus* viability with varying concentrations of L-His-Fe_3_O_4_ and Fe_3_O_4_. b-c. Viability of *S. aureus* **(b)** and *E. coli* **(c)** treated with H_2_O_2_ + L-His-Fe_3_O_4_. **(d)** Impact of L-His-Fe_3_O_4_ on *S. aureus* biofilm viability (Scale bar: 20 µm). **(e)** Crystal violet staining of *S. aureus* biofilm treated with L-His-Fe_3_O_4_. **(f)** Dry weight of *S. aureus* biofilm treated with L-His-Fe_3_O_4_. **(g)** ROS levels in *S. aureus* treated with L-His-Fe_3_O_4_. **(h)** Outer membrane permeabilization by L-His-Fe_3_O_4_. **(i)** Cytoplasmic membrane depolarization by L-His-Fe_3_O_4_. The data are presented as the means ± SD. *p < 0.05, **p < 0.01, ***p < 0.001, ****p < 0.0001.

Nanozymes with peroxidase (POD)-like enzyme activity can catalyze H_2_O_2_ to produce ∙OH, a ROS that induces bacterial cell death. The combination of nanozyme and H_2_O_2_ represents the primary mechanism of the antibacterial effect of nanozymes. To achieve a satisfactory antibacterial effect, we selected L-His-Fe_3_O_4_, which has high POD-like activity, for further investigation of its antibacterial performance in the presence of H_2_O_2_. To mitigate the toxicity of high H_2_O_2_ concentrations, we used a concentration much lower than the clinically effective range (0.5%–3%, wt%). The experimental results are shown in Figure 3B Following L-His modification, the antibacterial effect of Fe_3_O_4_ was significantly enhanced, with efficacy increasing proportionally with concentration (Figure 3a). Results in Figure 3b demonstrate that treating *S. aureus* with H_2_O_2_ or L-His-Fe_3_O_4_ (100 μg/mL) alone reduced bacterial counts by approximately 0.5 lg (CFU/mL) compared to the control. However, combining L-His-Fe_3_O_4_ (100 μg/mL) with H_2_O_2_ (L-His-Fe_3_O_4_/H_2_O_2_) reduced bacterial counts by 3.04 lg (CFU/mL). Additionally, the morphology of *S. aureus* after L-His-Fe_3_O_4_ treatment showed noticeable damage (Supplementary Figures S3, S4). Similar effects were observed in the elimination of other Gram-negative bacteria, such as *E. coli* (Figure 3c). Post L-His-Fe_3_O_4_ treatment, laser confocal microscopy revealed a significant increase in the proportion of dead cells within the *S. aureus* biofilm (Figure 3d), corresponding fluorescence intensity analysis of Figure 3d (Supplementary Figure S5). Along with a marked reduction in crystal violet staining and biofilm dry weight compared with the control (Figures 3e, f). The lipid oxidation fluorescent ratio-probe DCFH-DA indicated increased lipid ROS when *S. aureus* was treated with L-His-Fe_3_O_4_ (Figure 3g). ANS as a fluorescent probe can be used to detect membrane permeability. Fluorescence monitoring of ANS dye in *S. aureus* cells before and after L-His-Fe_3_O_4_ treatment revealed a significant increase in membrane permeability (Figure 3h). Using the membrane potential-sensitive probe DiSC3, we observed that L-His-Fe_3_O_4_ depolarized the *S. aureus* cell membrane, resulting in enhanced fluorescence (Figure 3i), indicating a reduction in membrane potential as DiSC3(5) was released into the solution upon membrane depolarization. This suggested that L-His-Fe_3_O_4_ interacted with the bacterial cell membrane, disrupting its integrity and leading to oxidative stress and cell death.

### 3.4 Biosafety studies in vitro and in vivo

**FIGURE 4:**
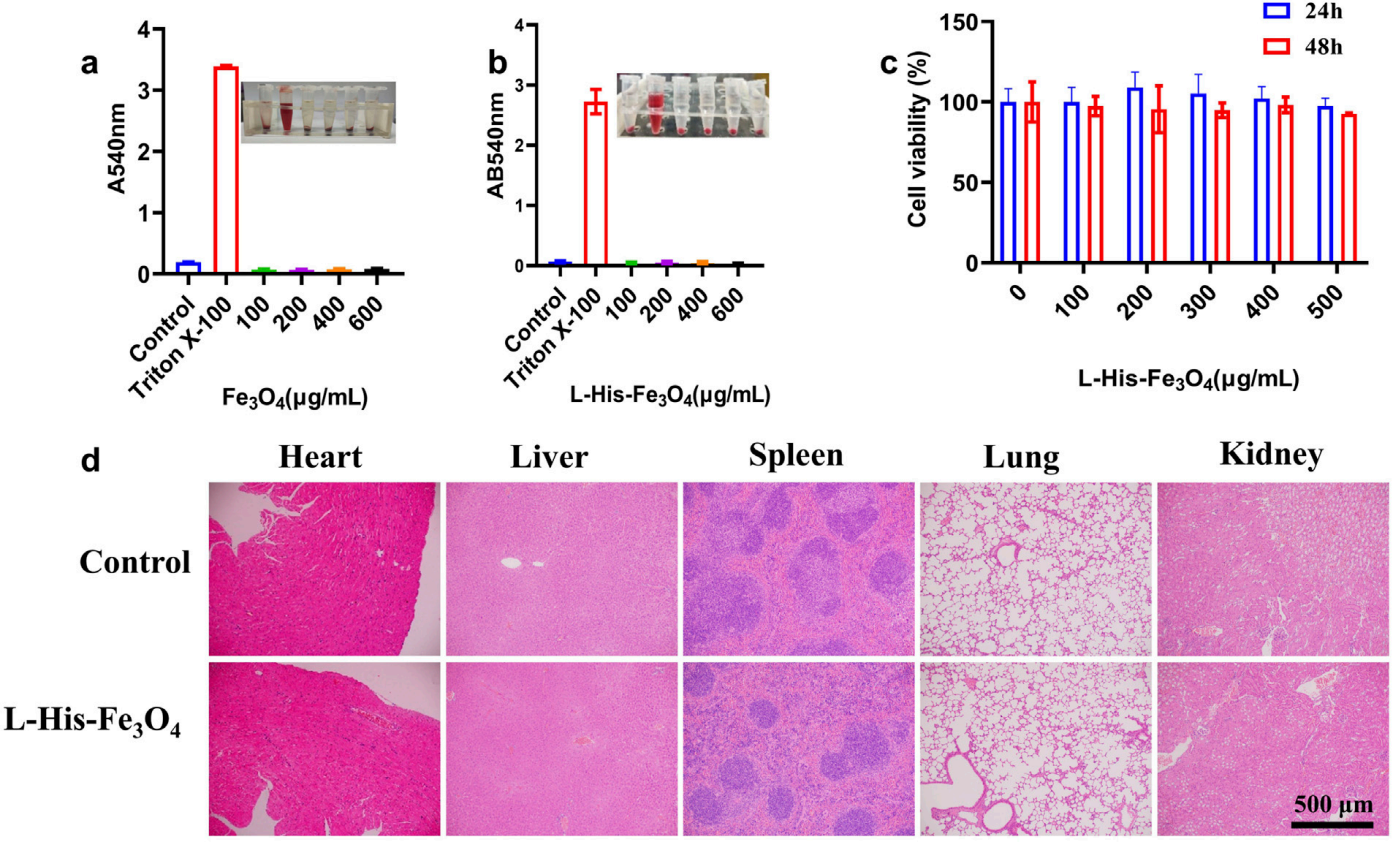
Biosafety assessment of L-His-Fe_3_O_4_. *In vitro* and *in vivo* toxicity evaluation of L-His-Fe_3_O_4_. **(a)** Hemolysis rate following treatment with Fe_3_O_4_ at varying concentrations. **(b)** Hemolysis rate following treatment with L-His-Fe_3_O_4_ at varying concentrations. **(c)** Cytotoxicity assessment results for L-His-Fe_3_O_4_. **(d)** Tissue sections of major organs treated with PBS and L-His-Fe_3_O_4_ (H&E staining; scale bar: 500 µm).

Good biocompatibility is essential for the *in vivo* and *in vitro* applications of L-His-Fe_3_O_4_. The hemolysis rate is a key indicator of material biocompatibility; thus, human red blood cells were used in a hemolysis test. As shown in Figures 4a, b, even at the highest concentrations of Fe_3_O_4_ and L-His-Fe_3_O_4_, the hemolysis rate remained below 1%. The viability of HaCaT cells incubated with various concentrations of L-His-Fe_3_O_4_ was assessed using a CCK-8 assay, revealing that cell viability remained above 95% across all concentrations (Figure 4c). To further evaluate the *in vivo* safety of L-His-Fe_3_O_4_, mice were treated with L-His-Fe_3_O_4_ for 7 days. After treatment, several major organs (heart, liver, spleen, lungs, kidneys) were collected, fixed in 4% neutral formaldehyde, and subjected to histopathological analysis with H&E staining. No lesions were detected in any of the groups, underscoring the biosafety of L-His-Fe_3_O_4_ (Figure 4d).

### 3.5 Evaluation of antibacterial properties *in vivo*

**FIGURE 5:**
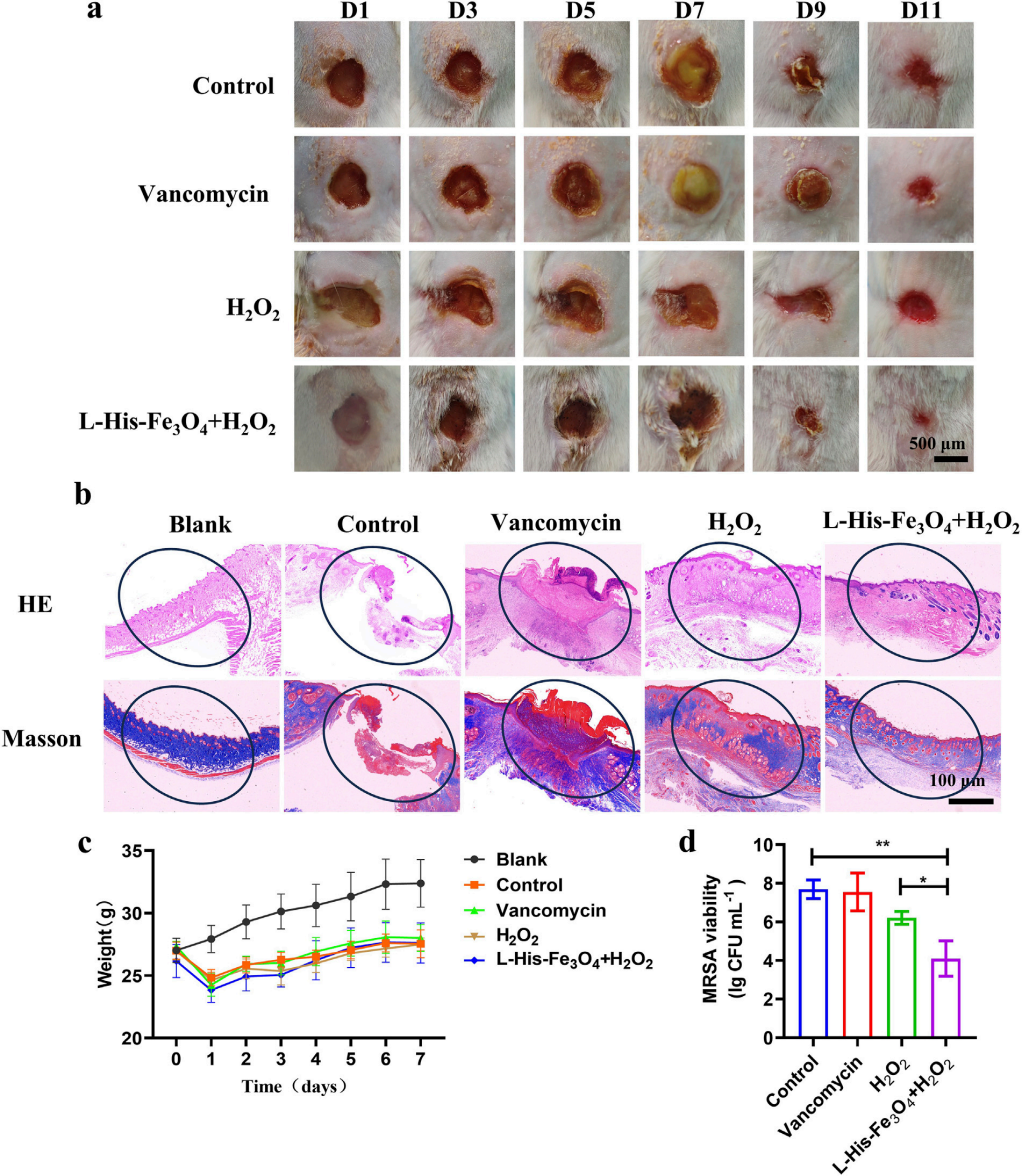
Application evaluation of bacterial infection in skin wounds. **(a)** Wound area of mice treated with PBS, Vancomycin, H_2_O_2_ and L-His-Fe_3_O_4_ over time. **(b)** H&E (top) and Masson staining (bottom) of infected tissues following different treatments. **(c)** Changes in body weight of mice over time with various treatments (n = 5) **(d)** Residual bacterial counts in wound tissue after different treatments. The data are presented as the means ± SD. *p < 0.05, **p < 0.01.

To further investigate the antibacterial efficacy of L-His-Fe_3_O_4_ and its potential to promote wound healing, we established a skin wound model of MRSA-infected mice with reference to the experimental method of Liu (Liu et al., 2023). Throughout treatment, wound changes were documented with photographs. As shown in Figure 5a, all experimental groups exhibited gradual scab formation and wound contraction. On day 7, the control and Vancomycin groups displayed severe purulent symptoms, whereas the H_2_O_2_ and L-His-Fe_3_O_4_ + H_2_O_2_ groups showed no purulence and began to scab. By day 9, scabs started forming in the control and Vancomycin groups, while those in the H_2_O_2_ and L-His-Fe_3_O_4_ + H_2_O_2_ groups began to peel off. On day 11, the wounds in the L-His-Fe_3_O_4_ + H_2_O_2_ group were significantly smaller than those in the other groups. To evaluate wound healing, H&E and Masson staining were performed on the wound tissues. As shown in Figure 5b, after 11 days of treatment, the control, Vancomycin, and H_2_O_2_ groups displayed incomplete epidermal layers, while the treatment group showed uniformly distributed collagen fibers. Additionally, we monitored the weight changes in each group during treatment (Figure 5c), finding no significant weight differences among the groups. On day 7, three mice from each group were selected, and the wound tissues were ground and subjected to plate counting; the results are shown in Figure 5d. The L-His-Fe_3_O_4_ + H_2_O_2_ treatment group exhibited significantly stronger antibacterial effects than the other groups, confirming that the treatment of infected wounds in mice with L-His-Fe_3_O_4_ + H_2_O_2_ is effective.

## 4 Discussion

With the increasing of antibiotic resistance in the world, the search for new antimicrobial agents has become a hot spot in today’s research (Dance, 2024). As a kind of antibacterial agent with high efficiency, selectivity and biocompatibility, nanozymes have received extensive attention (Labrag et al., 2023; Chakraborty et al., 2022). Among them, Fe_3_O_4_ nano-enzyme has become the focus of research because of its excellent performance and wide application prospect. In 2007, Gao et al. (2007) first identified magnetic iron oxide nanoparticles (Fe_3_O_4_ nanoparticles) with significant enzyme-like activity. Studies showed that Fe_3_O_4_ had a pH-dependent peroxide-like activity and catalase activity. In particular, Fe_3_O_4_ effectively catalyzes hydrogen peroxide to produce hydroxyl radicals in acidic conditions, which is antibacterial and kills tumor cells (Gao et al., 2016; Shi et al., 2018). Although Fe_3_O_4_ nanoenzymes have broad application prospects in biomedicine, there are still some challenges. Firstly, the synthesis method of Fe_3_O_4_ nanozymes need to be further optimized to improve their yield and stability. This study provides a convenient and rapid synthesis method for Fe_3_O_4_ at room temperature, supports large-scale production, and has important application prospects in the field of antibacterial.

The commonly used methods for preparing Fe_3_O_4_ include chemical coprecipitation, thermal decomposition, sol-gel, mechanical synthesis and oil phase coprecipitation (Yang et al., 2011). However, the existing preparation methods still have some defects: the preparation process is complicated, multiple parameters need to be controlled, and the preparation cycle is long (Wang et al., 2024). For example, hydrothermal synthesis of Fe_3_O_4_ often requires a high temperature of more than 200°C in a closed high-pressure reactor for 12 h (Gao et al., 2007), which requires high equipment and complex operation, which may cause certain pressure and safety hazards to the environment. We have synthesized L-His-Fe_3_O_4_ at room temperature by an improved method, L-His-Fe_3_O_4_ was quickly synthesized by stirring at room temperature for 1 min. TEM images show that L-His- Fe_3_O_4_ is a spherical particle with a diameter of about 20 nm, mainly composed of iron and oxygen elements (Figure 1). This method is simple to operate, the prepared iron oxide nanoparticles are of high quality and the end modification is flexible.

As a commonly used high reactive oxygen species, H_2_O_2_ has been widely used to prevent and control the infection of various pathogenic microorganisms. However, high doses of H_2_O_2_ can cause unnecessary damage to normal tissue, delaying healing. Fe_3_O_4_ nanozymes have peroxisase-like activity, which can promote the transformation of H_2_O_2_ to OH, and have great potential in tumor catalytic therapy and antimicrobial resistance (Zarandona et al., 2023; Anghel et al., 2014). Fe_3_O_4_ nanozymes with enzyme properties can effectively improve the antibacterial performance of H_2_O_2_, while avoiding the side effects of high concentration of H_2_O_2_, and become a promising antibacterial agent (Uwaya et al., 2020). We report a novel strategy for the rapid synthesis of Fe_3_O_4_ nanozymes by adding L-His. We demonstrated that L-His- Fe_3_O_4_ can be used to kill resistant bacteria such as *S*. *aureus* and *E. coli*, destroy biofilm (Figure 3). Animal experiments demonstrated the potential of L-His- Fe_3_O_4_ as a multifunctional material for wound infections (Figure 5).

In summary, this study demonstrates the synthesis of L-His-Fe_3_O_4_ using an ultrafast, low-cost method at room temperature. The addition of L-His significantly enhanced POD-like activity, endowing L-His-Fe_3_O_4_ with excellent antibacterial efficacy against *S. aureus* and *E. coli*. Following L-His modification, the antibacterial rate against *S. aureus* increased from 72.72% to 99.92%. The antibacterial effect relied on the POD-like activity of L-His-Fe_3_O_4_, which generated ∙OH to damage bacterial cell membranes. Cytotoxicity assessments via CCK-8 and red blood cell hemolysis tests confirmed high biosafety. *In vivo* experiments demonstrated a substantial therapeutic effect of L-His-Fe_3_O_4_ on bacteria-infected wounds. The synthesis method preserves the integrity of amino acids and can be applied to modify various functional groups, suggesting it as a potentially universal approach. This study offers a convenient and rapid synthesis method for Fe_3_O_4_, supporting large-scale production and presenting significant promise for future applications in the antibacterial field.

## Data Availability

The original contributions presented in the study are included in the article/Supplementary Material, further inquiries can be directed to the corresponding authors.
