# Supplementary material for: Ultrafast synthesis of L-His-Fe3O4 nanozymes with enhanced peroxidase-like activity for effective antibacterial applications
Source: Front Bioeng Biotechnol. 2025 Mar 28;13:1548025. doi: 10.3389/fbioe.2025.1548025 (PMC11985857; doi:10.3389/fbioe.2025.1548025)
Supplement: Supplementary file 1 [file DataSheet1.docx]

**Supporting Information**

**Ultrafast Synthesis of L-His-Fe₃O₄ Nanozymes with Enhanced Peroxidase-Like Activity for Effective Antibacterial Applications**

Ye Yuan ^#^, Yuan Liu ^#^, Zhipeng Shen, Huidan Wu, Lantian Meng, Xiaoxiao Guo, Bing Jiang*, Ling Fang*


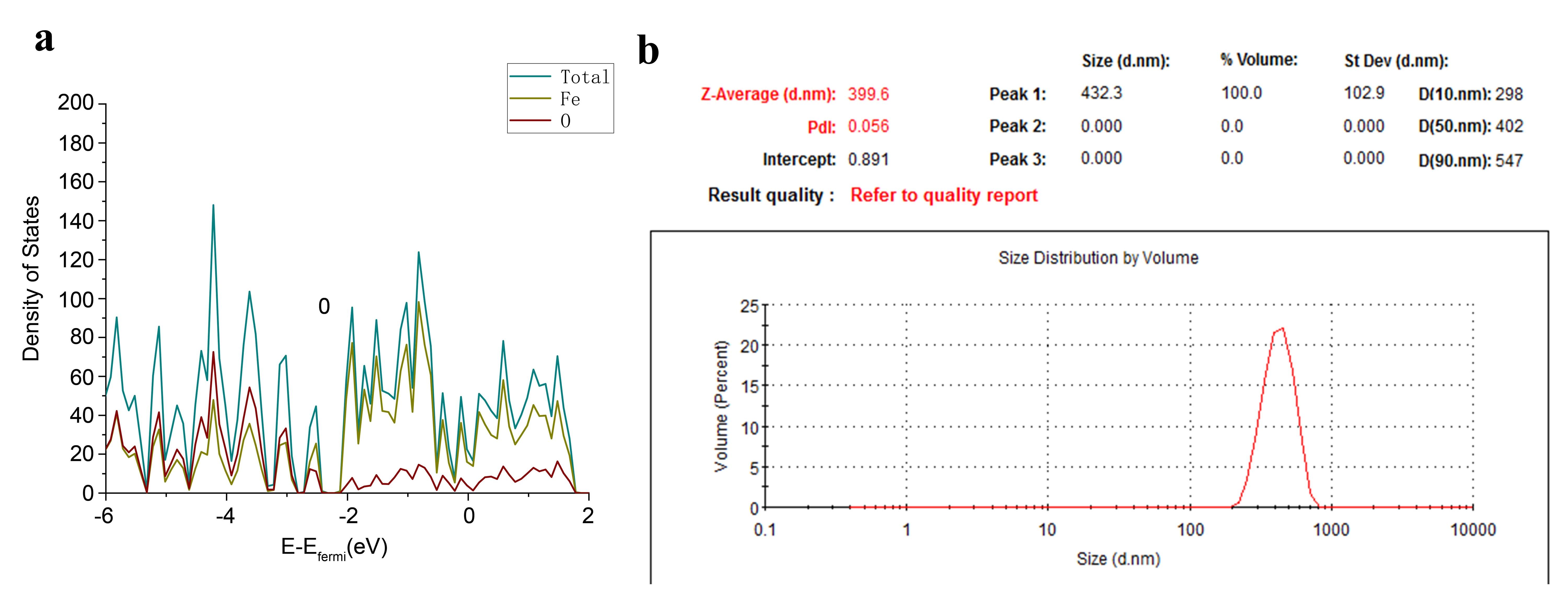


Figure S1. Density of states diagram of Fe_3_O_4_ (Figure S1a) and PDI of L-His-Fe_3_O_4_ (Figure S1b).


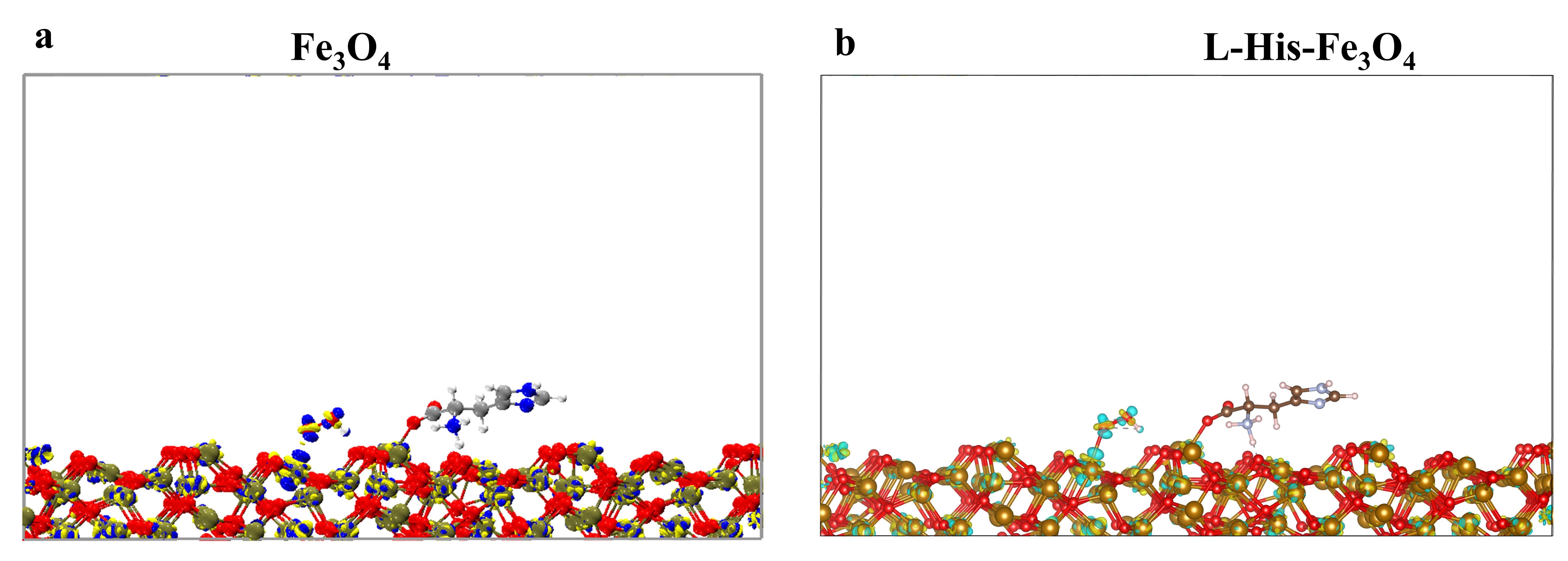


Figure S2. Differential charge density maps of Fe_3_O_4_  (Figure S2a) and L-His-Fe_3_O_4_ (Figure S2b).


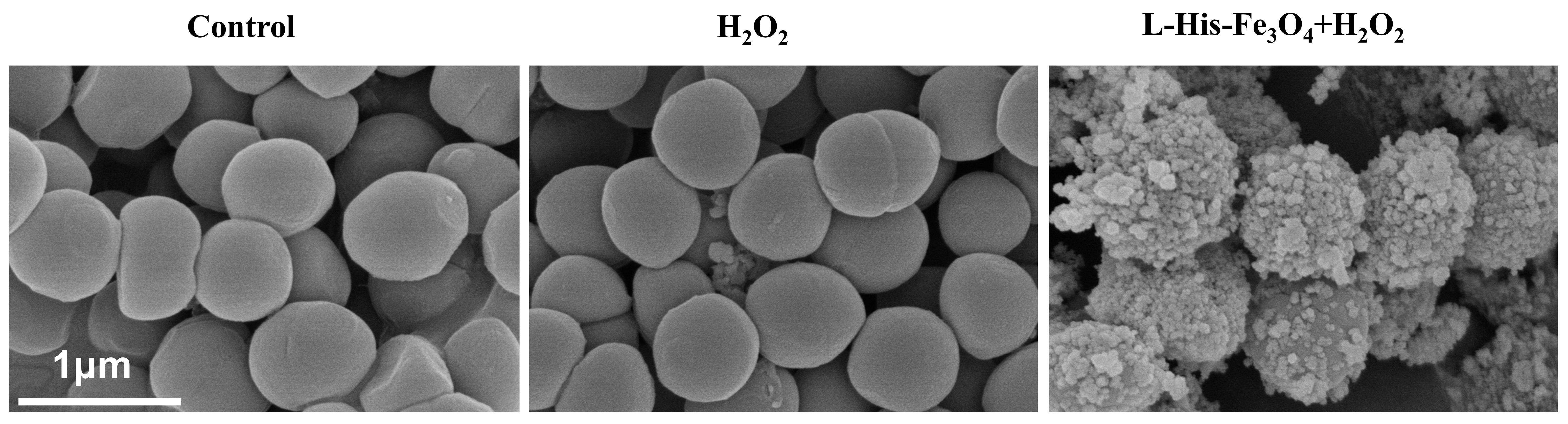


Figure S3. Typical SEM images of MRSA with different treatments.


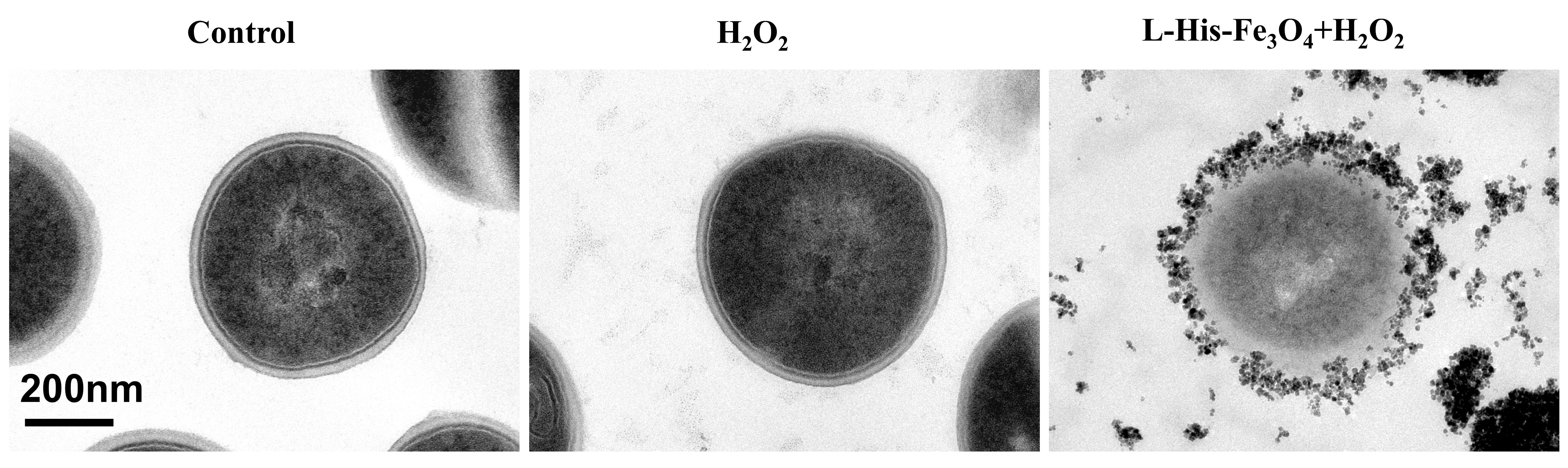


Figure S4. Typical TEM images of MRSA with different treatments.


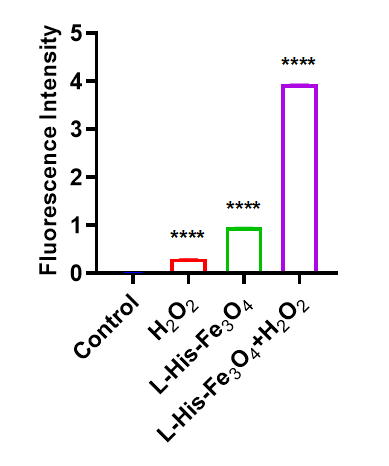


Figure S5. Corresponding fluorescence semi-quantitative analysis of Fig.3d.
